# Torsades de pointes and myocardial infarction following reversal of supraventricular tachycardia with adenosine: a case report

**DOI:** 10.31744/einstein_journal/2024RC0522

**Published:** 2024-03-21

**Authors:** Milena Ribeiro Paixão, Fernando Faglioni Ribas, Tarso Augusto Duenhas Accorsi, Karine De Amicis, José Leão de Souza

**Affiliations:** 1 Hospital Israelita Albert Einstein Sao Paulo SP Brazil Hospital Israelita Albert Einstein, Sao Paulo, SP, Brazil.

**Keywords:** Adenosine, Myocardial infarction, Tachycardia, supraventricular, Emergency service, hospital, Torsades de pointes

## Abstract

Adenosine is an antiarrhythmic drug that slows conduction through the atrioventricular node and acts as a coronary blood vessel dilator. This case report highlights two unusual life-threatening events following the use of adenosine to revert supraventricular tachycardia in a structurally normal heart: non-sustained polymorphic ventricular tachycardia and myocardial infarction. A 46-year-old woman presented to the emergency department with a two-hour history of palpitations and was diagnosed with supraventricular tachycardia. Vagal maneuvers were ineffective, and after intravenous adenosine administration, the patient presented with chest pain and hypotension. The rhythm degenerated into non-sustained polymorphic ventricular tachycardia and spontaneously reverted to sinus rhythm with ST elevation in lead aVR and ST depression in the inferior and anterolateral leads. The patient spontaneously recovered within a few minutes. Despite successful arrhythmia reversal, the patient was admitted to the intensive care unit because of an infarction without obstructive atherosclerosis. This report aims to alert emergency physicians about the potential complications associated with supraventricular tachycardia and its reversal with adenosine.

## INTRODUCTION

Adenosine is a nucleoside metabolite of adenosine triphosphate (ATP) dephosphorylation produced by myocytes and endothelial cells. The effects of adenosine on the cardiovascular system include vasodilatation, decreased atrioventricular node conduction, and antagonization of adrenergic stimulation.^(1)^ Synthetic adenosine is generally used for supraventricular tachycardia reversal in coronary function tests and coronary artery vasodilation during cardiac catheterization.^(2)^ Due to its short half-life, adenosine is usually safe, and there is a rapid normalization of the effects of receptor activation on vascular tissue. The most frequent symptoms observed, such as skin flushing, lightheadedness, nausea, sweating, nervousness, numbness, and feeling of impending doom, are unpleasant but not severe.^(3)^ Life-threatening cardiac adverse effects such as ventricular arrhythmia, atrioventricular block, prolonged asystole, hypotension, and ischemia are rare.^(4,5)^

This case report aims to highlight two unusual complications associated with the reversal of supraventricular tachycardia with adenosine: torsades de pointes and acute myocardial infarction.

## METHODS

### Case presentation

**Figure 1:**
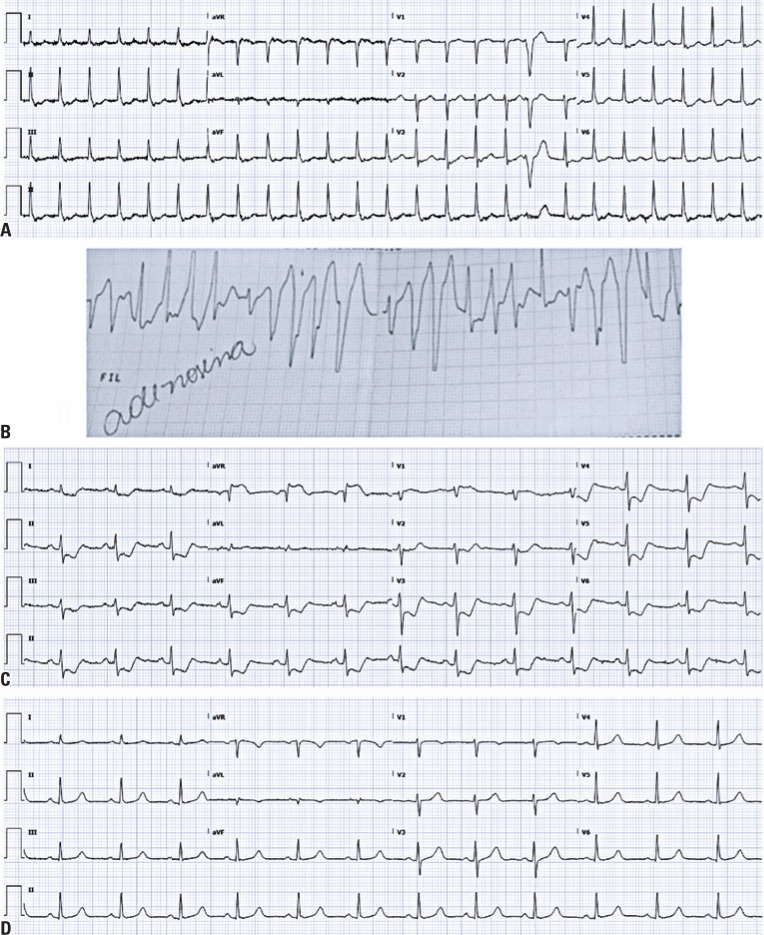
(A) Supraventricular tachycardia at admission; (B) Torsades des pointes after adenosine infusion. "Adenosine" handwritten on the electrocardiograph strip tracing is the Brazilian Portuguese word for adenosine; (C) Post-cardioversion electrocardiogram showing aVR ST elevation and diffuse ST depression; (D) Electrocardiogram 6 hours after admission

A 46-year-old woman was admitted to the emergency department with a two-hour history of palpitations and no signs of hemodynamic instability. The patient had a history of recurrent arrhythmias without other comorbidities or routine drug use. Electrocardiography on admission revealed supraventricular tachycardia (Figure 1A). Combined vagal maneuvers were ineffective in restoring sinus rhythm, and 6mg of intravenous adenosine was administered. Immediately after the venous infusion, the patient presented with chest pain, pale skin, diaphoresis, and hypotension. The rhythm degenerated to non-sustained polymorphic ventricular tachycardia (Figure 1B) and spontaneously reverted to sinus rhythm in less than 30 seconds. The second electrocardiogram showed ST-segment elevation in lead aVR and ST-segment depression in the inferior and anterolateral leads (Figure 1C). The patient recovered completely and spontaneously within a few minutes, with electrocardiogram normalization (Figure 1D). The troponin levels were elevated in a typical curve (Table 1). Invasive coronary angiography revealed no obstructive lesions. Echocardiography showed transient basal septal hypokinesia. The patient was discharged after two days.

**Table 1:** Myocardial necrosis troponin curve

Myocardial necrosis troponin curve						
Biomarker	p99	0h	1h	12h	24h	48h
Troponin T (pg/mL)	40	<40	300			
Troponin I (pg/mL)	52			5520	1700	1040

The work was approved by the Research Ethics Committee of *Hospital Israelita Albert Einstein* (CAAE: 53633921.0.0000.0071; # 5.149.637).

## DISCUSSION

Adenosine is a class I drug for the treatment of paroxysmal supraventricular tachycardia (PSVT).^(6)^ In this case, after adenosine infusion, heart monitoring revealed torsades de pointes, a rare but previously known phenomenon.^(7)^ Adenosine induces arrhythmia by several mechanisms including slowing ventricular rate during infusion and corresponding QT prolongation, inducing a compensatory response due to long sinus pause, shortening the effective refractory period of myocytes, and increasing ventricular automaticity by reflex catecholamine release.^(8)^

The return to sinus rhythm was accompanied by an aVR ST-segment elevation and diffuse ST-segment depression, suggesting an acute lesion of the main or proximal left anterior descending coronary artery. The initial hypothesis was that this could be due to repolarization changes mimicking myocardial ischemia after PSVT.^(9)^ However, the patient's chest pain started after PSVT reversal and was associated with hemodynamic instability, elevation of necrosis markers in a typical curve, and segmental changes on echocardiography, confirming an acute myocardial infarction.

Paroxysmal supraventricular tachycardia is recognized as a potential cause of troponin elevation, which may not necessarily indicate myocardial necrosis, but rather myocardial damage. The pathophysiological mechanism may be associated with diastolic shortening, potentially leading to subendocardial ischemia.^(10)^ Factors such as older age, chest pain at presentation, lower diastolic blood pressure, prolonged tachycardia, elevated heart rate during PSVT, and impaired left ventricular systolic function have been reported to influence troponin levels in patients with PSVT.^(10,11)^ Additionally, high levels of cortisol and catecholamines may be linked to increased troponin levels, suggesting that stress contributes to myocardial damage.^(12,13)^ Takotsubo syndrome was another potential diagnosis for this patient; however, the echocardiogram did not reveal any transient abnormalities in the left ventricular wall motion that would extend beyond the perfusion territory of a single epicardial coronary artery, and troponin levels were higher than those typically observed in Takotsubo syndrome.^(13,14)^

In this case, the absence of coronary lesions on angiography, transient cardiovascular impairment, elevated troponin levels, and lack of criteria for Takotsubo syndrome strengthened the hypothesis of a coronary vasospasm-induced myocardial infarction. Some cases of adenosine-induced vasospasm were reported in the literature during pharmacological stress for scintigraphy,^(15,16)^ positron emission tomography,^(17)^ angiotomography,^(18)^ and cardiac catheterization.^(19)^ There are two reports of vasospasm caused by adenosine during the treatment of supraventricular tachycardia.^(20,21)^

There are some possible explanations for adenosine-induced vasospasm. Four adenosine receptors have been identified: A1, A2A, A2B, and A3.^(7)^ The A1 receptor reduces cAMP production; thus, it is responsible for a transient atrioventricular block, the desired effect in the treatment of supraventricular tachycardia; however, it can prevent relaxation of the coronary artery smooth muscle when expressed in this tissue. The A2 receptor increases cAMP production, thereby activating an ATP-dependent potassium channel, relaxing smooth muscle cells, and causing the desired vasodilatory effect in functional tests used to assess coronary diseases.^(19)^ The smooth muscle cells have different concentrations of both receptors. An imbalance favoring stimulation of the A1 receptor could contribute to coronary spasm. Interruption of the vasodilatory effect of adenosine after its half-life, which fails to counterbalance the vasoconstrictor mechanisms, could also contribute to this phenomenon.^(16)^ The presence of potentially vasoconstrictive substances, such as catecholamines and endothelin-1, may also contribute to vasospasm.^(17,18)^ The endothelium plays a central role in balancing these substances and its dysfunction may be related to the paradoxical adenosine effect.^(22)^

The occurrence of torsades de pointes and myocardial infarction immediately after adenosine administration led us to presume that the drug precipitated these events. Nonetheless, coronary angiography performed after the chest pain and hemodynamic instability had improved did not reveal vasospasm. Consequently, without evidence of vasospasm during this test, we could not definitively determine the exact mechanism responsible for the myocardial injury.

## CONCLUSION

This case report highlights the complex cardiovascular effects of adenosine in the treatment of paroxysmal supraventricular tachycardia, particularly the rare but serious potential to induce torsades de pointes and acute myocardial infarction. This case adds to the existing evidence on the cardiovascular risks associated with adenosine and emphasizes the importance of being prepared to manage potential complications during its administration.
